# Correction to: ‘Wavelength-dependent effects of artificial light at night on phytoplankton growth and community structure’ (2021) by Diamantopoulou *et al.*

**DOI:** 10.1098/rspb.2022.1705

**Published:** 2022-11-30

**Authors:** Christina Diamantopoulou, Eleni Christoforou, Davide M. Dominoni, Eirini Kaiserli, Jakub Czyzewski, Nosrat Mirzai, Sofie Spatharis

*Proc. R. Soc. B* **288**, 20210525. (Published online 23 June 2021). (https://doi.org/10.1098/rspb.2021.0525)

We apologize for any inconvenience regarding the presentation of results on the *Tetraselmis suecica* growth rate. We hereby make the following three corrections in the Results, figure 1*a* and electronic supplementary material, table S1.

**Figure:**
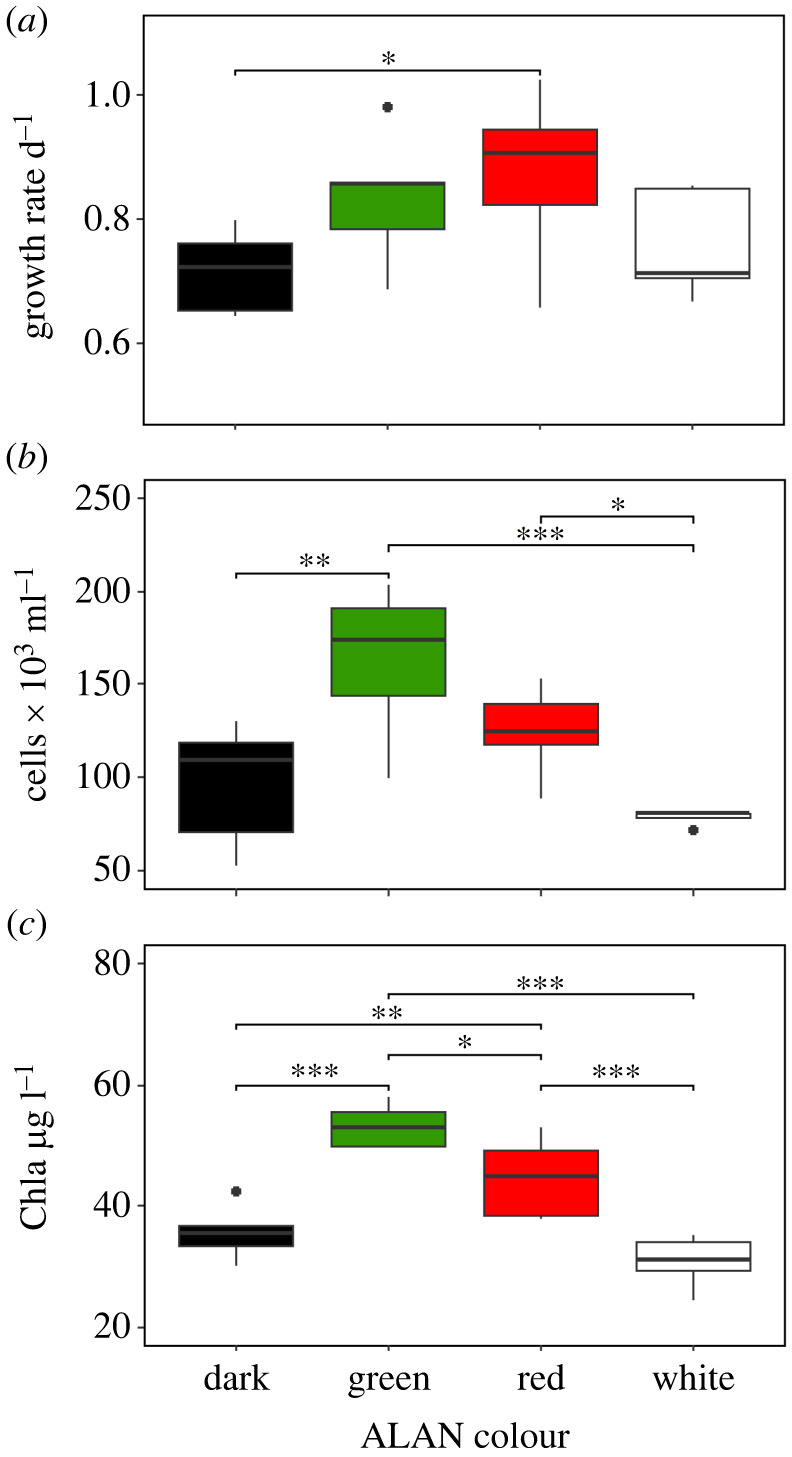

Correction 1:

The first paragraph of the Results should read: The ALAN treatment did not have an effect on the growth rate of *Tetraselmis suecica* (*F*_3,16_ = 2.275, *p* = 0.119) (figure 1*a*). *t*-test pairwise comparisons show a significant difference of the red ALAN treatment from the dark (electronic supplementary material, table S1).

Correction 2:

Correction of panel (*a*) in figure 1.

Correction 3: Correction of growth rate in electronic supplementary material, table S1.

**Table:** 

pairwise comparison	growth rate	cells	Chl a
dark–green	−0.117 (0.095)	−65867 (0.002)	−17.55 (<0.000)
dark–red	−0.155 (0.032)	−28200 (0.149)	−9.01 (0.010)
dark–white	−0.042 (0.538)	17933 (0.350)	4.83 (0.137)
green–red	0.062 (0.178)	37667 (0.060)	8.54 (0.014)
green–white	0.076 (0.269)	83800 (0.000)	22.38 (<0.000)
red–white	0.114 (0.105)	46133 (0.025)	13.85 (<0.001)

